# Protective potential of outer membrane vesicles derived from a virulent strain of *Francisella tularensis*

**DOI:** 10.3389/fmicb.2024.1355872

**Published:** 2024-03-12

**Authors:** Ivona Pavkova, Jan Bavlovic, Klara Kubelkova, Jiri Stulik, Jana Klimentova

**Affiliations:** Department of Molecular Pathology and Biology, Military Faculty of Medicine, University of Defence, Hradec Kralove, Czechia

**Keywords:** *Francisella tularensis*, FSC200, outer membrane vesicles, host-pathogen interaction, vaccination

## Abstract

*Francisella tularensis* secretes tubular outer membrane vesicles (OMVs) that contain a number of immunoreactive proteins as well as virulence factors. We have reported previously that isolated *Francisella* OMVs enter macrophages, cumulate inside, and induce a strong pro-inflammatory response. In the current article, we present that OMVs treatment of macrophages also enhances phagocytosis of the bacteria and suppresses their intracellular replication. On the other hand, the subsequent infection with *Francisella* is able to revert to some extent the strong pro-inflammatory effect induced by OMVs in macrophages. Being derived from the bacterial surface, isolated OMVs may be considered a “non-viable mixture of *Francisella* antigens” and as such, they present a promising protective material. Immunization of mice with OMVs isolated from a virulent *F. tularensis* subsp. *holarctica* strain FSC200 prolonged the survival time but did not fully protect against the infection with a lethal dose of the parent strain. However, the sera of the immunized animals revealed unambiguous cytokine and antibody responses and proved to recognize a set of well-known *Francisella* immunoreactive proteins. For these reasons, *Francisella* OMVs present an interesting material for future protective studies.

## 1 Introduction

The release of membrane vesicles is a phenomenon common to organisms of all kingdoms (Gill et al., 2018). The bacterial vesicles have shown great potential in various biomedical applications due to their unique composition and strong ability to interact with the immune system (Baker et al., 2014; Gnopo et al., 2017; Krishnan et al., 2022). They play a considerable role in the development of new vaccine platforms (Acevedo et al., 2014; Gnopo et al., 2017; Gerritzen et al., 2019; Wang et al., 2020), where their self-adjuvant abilities and non-replicative nature present notable advantages over convenient live attenuated or subunit vaccines. Moreover, they have also recently been studied as promising agents in cancer immunotherapy (Park et al., 2021) and as advanced drug delivery systems (Liu et al., 2021).

The vesicles derived from Gram-negative bacteria are denoted as outer membrane vesicles (OMVs). Naturally produced OMVs are nanosized particles formed by bulging and separation of a portion of the bacterial outer membrane closing inside contents of the periplasm (Avila-Calderón et al., 2021). OMVs thus mimic the composition of the parent bacterium surface and periplasm but they are not viable and replicative. Depending on the bacterial environment and actual conditions bacteria are capable of enriching particular proteins or other molecules in the released OMVs and responding thus to the current situation (Bonnington and Kuehn, 2014; Klimentova et al., 2019). OMVs serve a plethora of functions in bacterial physiology, like intercellular communication, secretion of biologically active molecules, nutrient acquisition, or stress response, and they play important roles also in the pathology of bacterial infection, e.g., in the immune response modulation or depletion of host-generated antibacterial molecules (Manning and Kuehn, 2013; Kaparakis-Liaskos and Ferrero, 2015; Caruana and Walper, 2020).

OMVs have already shown their protective potential against a wide spectrum of bacteria (Micoli and MacLennan, 2020). The best known is the OMV-based vaccine against *Neisseria meningitidis* (Gorringe and Pajón, 2012; O’Ryan et al., 2014) but intensive attention is also being given to OMV-based preparations against *Salmonella* (Micoli et al., 2018), *Klebsiella pneumoniae* (Lee et al., 2015), *Vibrio cholerae* (Adriani et al., 2018), *Haemophilus influenzae* (Roier et al., 2012), *Yersinia pestis* (Wang et al., 2020), and many others. The strong capacity of OMVs to induce the immune system is given by their small size and the fact, that they carry a rich mixture of miscellaneous pathogen-associated molecular patterns, which makes them attractive for antigen-presenting cells (Micoli and MacLennan, 2020). However, the protective effect of OMVs is usually outweighed by a strong pro-inflammatory response, which can turn fatal and limit their use. For the most part, it is accredited to the presence of toxic lipopolysaccharide (LPS) and its strong stimulation of Toll-like receptors (TLR) signaling pathways. Detoxification of the LPS by its removal or genetic manipulations thus presents the main methods of OMVs engineering (Chen et al., 2016; Wang et al., 2020).

It has been described previously that *Francisella tularensis*—an intracellular bacterium and a causative agent of the severe disease tularemia—produces OMVs of unusual tubular shape, that are highly enriched in immunomodulatory proteins and virulence factors (McCaig et al., 2013; Klimentova et al., 2019, 2021). The *Francisella* OMVs induce a strong pro-inflammatory response in macrophages which is in deep contrast with the overall “silencing” effect of the parent bacterium and are also involved in the entry of the bacterium into the host cells (Pavkova et al., 2021). The LPS of *F. tularensis* has unusual biological activity caused by its atypical structure, due to which it fails to act as a pro-inflammatory endotoxin with no or very weak potency to stimulate TLR4 (and TLR2) and cytokine production (Barker et al., 2006; Dueñas et al., 2006). This can be taken as an advantage regarding the potential use of *Francisella* OMVs as a protective agent and for this reason, the *Francisella* LPS has been genetically introduced in the OMVs of hyper vesiculating strains of *Y. pestis* (Wang et al., 2020) or *E. coli* (Chen et al., 2016). The presence of the atypical LPS is also critical for the virulence of *Francisella* and the tubular shape of its OMVs (Bavlovic et al., 2023).

At present, scant effort has been dedicated to testing the protective potential of *Francisella* OMVs. In *F. novicida*, a species related to *F. tularensis* but with much lower virulence in humans, the OMVs vaccination on mice showed moderate protection against the parent strain (McCaig et al., 2013). In *F. noatunensis*, a fish pathogen, the OMVs showed some potential in zebrafish (Brudal et al., 2015) but failed in tilapia and Atlantic cod (Mertes et al., 2021). Regarding *F. tularensis*, outer membrane fraction isolated from *F. tularensis* live vaccine strain (LVS) was used as an acellular subunit vaccine with 50% survival efficiency against the most virulent *F. tularensis* subsp. *tularensis* SchuS4 strain (Huntley et al., 2008). Similarly, efforts were made to immunize mice with LVS or SchuS4 membranes encapsulated in poly (lactic-co-glycolic) acid nanoparticles which provided a promising protective effect against the infection (Post et al., 2017).

In the current study, we evaluated the potential protective effect of OMVs isolated from a virulent *F. tularensis* subsp. *holarctica* strain FSC200 *in vitro* on macrophages and *in vivo* in mice. On the cellular level, we noticed that OMVs treatment induces the uptake of the bacteria into macrophages and suppresses their intracellular multiplication. On the other hand, the strong pro-inflammatory effect raised by OMVs treatment was partially dampened by the following infection with *Francisella*. After immunization of mice with *Francisella* OMVs we have observed significant but weak protection against the infection with the parent strain. However, the sera of the immunized animals revealed unequivocal cytokine and antibody response and proved to recognize a set of well-known *Francisella* immunoreactive proteins.

## 2 Materials and methods

### 2.1 Bacteria and cultivation

*Francisella tularensis* subsp. *holarctica* strain FSC200 was provided by Åke Forsberg (Swedish Defense Research Agency, Umeå, Sweden). Bacteria were cultivated on McLeod agar plates supplemented with bovine hemoglobin and IsoVitaleX (Becton Dickinson) at 37°C for 24 h. Brain heart infusion (BHI; Becton Dickinson) was prepared according to the manufacturer’s instructions, pH was adjusted to 6.8, and it was sterile-filtered.

### 2.2 Outer membrane vesicles isolation

Outer membrane vesicles were prepared as described earlier (Klimentova et al., 2019) from large-scale cultivations (2–6 L). Briefly, bacteria from agar plates were inoculated in BHI for pre-cultivation (ca 20 h, 37°C, 200 rpm). After centrifugation (6,000 × g, 15 min, 25°C), the desired volume of suspension of OD_600_ = 0.1 was prepared and cultivated for 14–16 h. Bacteria were pelleted (10,000 × g, 20 min, 4°C), and the supernatants were sterilized by filtration through a 0.22-mm vacuum-driven filter and concentrated using Amicon Stirred Ultrafiltration Cell (Millipore) through a membrane of regenerated cellulose with 100 kDa cutoff (Millipore). OMVs were pelleted (100,000 × g, 90 min at 4°C), resuspended in 45% OptiPrep (Sigma-Aldrich) in 10 mM HEPES/0.85% NaCl, pH 7.4 (HEPES buffer), and overlaid with a step OptiPrep gradient of 40–20%. The gradient was centrifuged (100,000 × g, overnight, 4°C) in a swinging bucket rotor. After centrifugation, the top fractions containing opaque white bands were collected, diluted 8× with HEPES buffer, and centrifuged (100,000 × g, 2 h, at 4°C). The supernatant was removed and the pellet was washed again to remove the residual OptiPrep. The final pellet was suspended in physiological saline and the protein concentration was determined with the Micro BCA™ Protein Assay Kit (Pierce).

### 2.3 Murine bone marrow macrophages isolation

Murine bone marrow macrophages (BMMs) were isolated from femurs and tibias of six-to-ten-week-old female BALB/c mice according to a described protocol (Celli, 2008). Briefly, cells flushed from the bone marrow were placed in bacteriological Petri dishes and differentiated in Dulbecco’s Modified Eagle Medium (DMEM, Invitrogen) with 10% fetal bovine serum, 20% (v/v) L929-conditioned medium (as a source of macrophage colony-stimulating factor), and 50 U/mL penicillin/50 mg/mL streptomycin (only for the first 3 days of cultivation). After 6 days of differentiation, BMMs were seeded on tissue culture-treated multi-well plates at the desired densities as further specified in the relevant assay procedure.

### 2.4 Intracellular replication of bacteria in OMV-treated macrophages

The differentiated BMMs were seeded at a concentration of 1 × 10^5^ cells/well in 24-well plates and adhered overnight at 37°C and 5% CO_2_. The supernatant was discarded and the cells were treated with OMVs (0.5 μg per well) or IFN-γ (5 U/mL) for 24 h, untreated BMMs were used as control. The next day medium was discarded and BMMs were infected with *F. tularensis* FSC200 at multiplicity of infection (MOI) 50. To synchronize the infection, the plates were briefly centrifuged (400 × g, 5 min) and incubated at 37°C and 5% CO_2_ for 30 min. To remove the extracellular bacteria cells were rinsed twice with phosphate-buffered saline (PBS) and DMEM with gentamicin (5 μg/mL) was added for a further 30 min, then it was replaced by fresh DMEM. At the indicated time intervals BMMs were lysed with 0.1% sodium deoxycholate (5 min on ice) and intracellular bacteria were enumerated by plating the serial dilutions. Three replicates of each condition were evaluated in one experiment and the experiment was repeated three times with comparable results.

### 2.5 Evaluation of cytokine release in BMMs treated with OMVs and infection

BMMs at a concentration of 1 × 10^5^ cells/well were adhered on 24-well plates overnight. The cells were treated with OMVs (0.5 μg per well) for 4 h, the supernatants were collected, designed as *t* = 0 h, and stored at −80°C. The same cells were then immediately infected with FSC200 at MOI = 50, briefly centrifuged (400 × g, 5 min) and incubated for 1 h. Then they were washed twice with PBS to remove the majority of extracellular bacteria and further cultivated in fresh DMEM. At *t* = 4 h and *t* = 24 h post-infection, the supernatants were collected. The second group of cells was first infected with FSC200 (as above), after 4 h post-infection the supernatants were collected (*t* = 0 h), and then they were treated with OMVs (as above). The third and fourth groups were treated only by OMVs or by infection, respectively, and untreated uninfected cells were used as controls.

The quantities of secreted cytokines or chemokines were measured using mouse ELISA kits against IL-6 (# BMS603-2), TNF-α (# BMS607-3), IL-12p70 (# BMS6004), IFN-β (# 424001), and CXCL-1 (# EMCXCL1) (Thermo Fisher Scientific) according to the manufacturer’s instructions. The absorbance was measured at 450 nm on a Paradigm detection platform (Beckman Coulter). The resulting concentrations were determined from standard calibration curves. Three replicates of each condition were evaluated in one experiment and the experiment was repeated twice with comparable results.

### 2.6 Western blot analysis of treated macrophages

BMMs at a concentration of 2 × 10^6^ cells/well were adhered on 6-well plates overnight. The cells were treated for 24 h with the following stimuli: OMVs (10 μg per well), FSC200 (MOI = 50), LPS from *E. coli* O55:B5 (Sigma-Aldrich; 500 ng/mL), or IL-4 (Sigma-Aldrich; 20 ng/mL). Untreated BMMs were used as control. The cells were then washed twice with PBS and lysed with RIPA buffer for 30 min on ice and overnight at −80°C. The lysates were cleared by centrifugation (14,000 × g, 10 min, 4°C), and protein concentrations were determined by bicinchoninic acid assay. Samples were separated by 8% SDS-PAGE and electroblotted onto polyvinylidene difluoride (PVDF) membrane (Boehring). The proteins were immunodetected by primary rabbit polyclonal antibodies against alpha-tubulin 1A (#PA5-22060), iNOS (#PA3-030A), and arginase 1 (#PA5-29645, all Invitrogen), and polyclonal HRP-conjugated swine anti-rabbit immunoglobulins (Dako Cytochrom) were used as the secondary antibody. The reaction was visualized by SuperSignal™ West Femto Maximum Sensitivity Substrate on the iBright^™^ FL1000 Imaging System (both Thermo Fisher Scientific) and quantified with iBright Analysis Software. Local background corrected volumes were used and the values were normalized using alpha tubulin 1A bands as the house-keeping protein.

### 2.7 *In vivo* protection studies of OMVs against infection

The BALB/c mice were used for OMVs protection experiments. The animals were maintained with water and powder chow provided *ad libitum*. Mice were anesthetized with ketamine 50 mg/mL and xylazine 20 mg/mL (both Bioveta, Ivanovice na Hane, Czech Republic) according to the manufacturer’s manual and vaccinated intranasally (i.n.) or intraperitoneally (i.p.) with 15 μg of OMVs in physiological saline, control mice were given only the saline. Fourteen days after the first immunization the mice were boosted with the same, 42 days after the first immunization they were challenged with 100 CFU of *F. tularensis* FSC200 per mouse (by the same route of infection as the vaccination) and observed for survival. On days 14 and 42 after the first immunization, respectively, three vaccinated mice were sacrificed for the collection of immune sera. The mice were killed by cervical dislocation, blood was collected, and serum was prepared by clotting at room temperature. The sterility of the serum was confirmed by plating on McLeod agar. Nonimmune control sera were acquired from physiological saline-treated mice without any exposure to OMVs.

### 2.8 Determination of antibody isotypes and cytokine profiles in immune sera

The immunoglobulin subclasses of serum samples of control or vaccinated BALB/c mice were determined using Quantibody Mouse Immunoglobulin Isotype Array (RayBiotech Life) screening the following set of eight mouse immunoglobulin subclasses (IgG1, IgG2a, IgG2b, IgG3, IgA, IgD, IgE, and IgM) plus two light chain types (kappa and lambda). The evaluation was performed following the manufacturer’s instructions. The arrays were scanned with a laser scanner GenePIX 4000 Microarray Scanner (Axon Instrument) for fluorescence intensity. The image analysis was performed in AGScan by subtracting the local background from the fluorescence intensity of each spot. The obtained data were manually checked for the presence of non-specific fluorescence and spot errors, and suspicious spots were removed from the analysis. The data thus obtained was analyzed using microarray analysis software (GenePix Pro 4.1, Axon Instrument) and converted to concentrations using the H20 OV Q-Analyzer v8.20.4 program (RayBiotech).

The cytokine profiles of the murine sera were analyzed using fluorescence-based multiplex Quantibody ELISA microarray chip (RayBiotech) screening the following cytokines: G-CSF, GM-CSF, IL-1α, IL-1β, IL-2, IL-3, IL-4, IL-5, IL-6, IL-7, IL-9, IL-10, IL-12p70, IL-13, IL-15, IL-17, IL-21, IL-23, IFN-γ, and TNF-α. The evaluation was performed following the manufacturer’s protocol. The scanning protocol and image analysis are the same as for the antibody isotypes.

### 2.9 2D gel electrophoresis and detection of immunoreactive proteins in mice sera

Whole-cell lysate of FSC200 separated by 2D SDS-PAGE was used as the antigen for the detection of immunoreactive proteins recognized by the murine immune sera. Bacteria cultivated in BHI were washed twice in ice-cold PBS. Then, they were resuspended in 50 mM ammonium bicarbonate (ABC) supplemented with protease inhibitors cocktail Complete EDTA-free (Roche Diagnostics) and disrupted in French pressure cell (Thermo IEC) by two passages at 1,600 psi. The lysate was treated with benzonase (Sigma-Aldrich), 150 U/mL, for 10 min on ice. Unbroken cells were removed by centrifugation (12,000 × g, 20 min, 4°C), and the supernatant was filter sterilized through a Millex-GP Syringe Filter Unit (0.22 μm, polyethersulfone, Millipore). Protein concentration was determined by bicinchoninic acid assay.

The lysate (100 μg) was solubilized in a rehydration buffer containing 9 M urea, 4% CHAPS, 70 mM dithiothreitol, and 5% v/v carrier ampholytes, pH 9–11 (Sigma-Aldrich) and separated by isoelectric focusing on 7 cm gradient pH 3–10 Immobiline DryStrip gels (GE Healthcare, Uppsala, Sweden) and 12% SDS-PAGE using Multiphor II and Protean II Multi-cell electrophoresis system (Bio-Rad). Coomassie G-250 staining was used to visualize proteins on the gels for the purpose of mass spectrometry. The rest of the gels were electroblotted onto the PVDF membrane (Boehring). Immunoreactive proteins on the membranes were detected by incubation with control or immune sera (pooled from three mice) diluted 1:100 in 4% skimmed milk in 0.1% Tween TBS overnight at 4°C. Polyclonal HRP-conjugated goat anti-mouse immunoglobulins (Dako Cytochrom) were then used for secondary antibody detection. The reaction was visualized using the BM Chemiluminescence Blotting Substrate kit (Boehring) on CL-XPosure films (Pierce, Rockford, IL). Each immunoblot experiment was conducted in technical duplicate and the membranes post-detection were silver stained.

### 2.10 In gel digest

Selected spots were excised from the Coomassie-stained gels, destained in 100 mM ABC in 50% acetonitrile (ACN), washed briefly in methanol, equilibrated in 100 mM ABC, and dehydrated in ACN. The proteins were reduced by 10 mM dithiothreitol in 100 mM ABC (30 min, 56°C) and alkylated by 55 mM iodoacetamide in 100 mM ABC (20 min, RT, dark). The gels were then equilibrated in 100 mM ABC, dehydrated in ACN, and digested by 0.1 μg of sequencing grade trypsin (Promega) in 50 mM ABC (37°C, overnight). Supernatants were collected, the gel pieces were overlaid by 5% formic acid, and extracted for 20 min (RT), then the same volume of 80% ACN was added and extraction continued for a further 20 min. The extracts were combined with the corresponding supernatants, dried in a vacuum, and desalted using Cleanup C18 Pipette Tips (Agilent) according to the manufacturer’s instructions.

### 2.11 LC–MS/MS analysis and database search

LC–MS/MS analysis was performed on the Ultimate 3000 RSLCnano System (Dionex) coupled online through Nanospray Flex ion source with Q-Exactive mass spectrometer (Thermo Scientific). Peptides were dissolved in 2% ACN/0.05% trifluoroacetic acid and loaded onto a capillary trap column (C18 PepMap100, 3 μm, 100 Å, 0.075 × 20 mm; Dionex) by 5 μL/min of 2% ACN/0.05% trifluoroacetic acid for 5 min. Then they were separated on the capillary column (C18 PepMap RSLC, 2 μm, 100 Å, 0.075 × 150 mm; Dionex) by step linear gradient of mobile phase B (80% ACN/0.1% formic acid) over mobile phase A (0.1% formic acid) from 4 to 36% B in 19 min and from 36 to 55% B in 6 min at flow rate of 300 nL/min. The column was kept at 40°C and the eluent was monitored at 215 nm. The spraying voltage was 1.75 kV and the heated capillary temperature was 275°C. The mass spectrometer was operated in the positive ion mode performing survey MS (at 350–1,650 m/z) and data-dependent MS/MS scans of 7 most intense precursors with a dynamic exclusion window of 23 s and isolation window of 1.6 Da. MS scans were acquired with a resolution of 70,000 from 10^6^ accumulated charges at a maximum fill time of 100 ms. The normalized collision energy for HCD fragmentation was 27 units. MS/MS spectra were acquired with a resolution of 17,500 from 10^5^ accumulated charges at a maximum fill time of 100 ms.

Proteins were identified using the SequestHT search engine within Proteome Discoverer v. 2.4.1.15 (Thermo Fisher Scientific). The reference proteome set of *F. tularensis* subsp. *holarctica* strain FSC200 was downloaded from UniProt in May 2018 (1,420 sequences) and Proteome Discoverer implemented cRAP database (118 sequences) was used for the identification of common contaminants. The search parameters were: digestion with trypsin (two missed cleavages allowed), minimum peptide length: 6, fixed modification: carbamidomethylation of cysteine; variable modifications: oxidation of methionine, formylation and Met-loss of protein N-term, mass tolerances of precursor and fragment ions: 10 ppm and 0.02 Da, respectively. The search results were processed in the consensus workflow, and the filter of two peptides per protein with an FDR threshold of 0.05 was applied. The mass spectrometry proteomics data have been deposited to the ProteomeXchange Consortium via the PRIDE (Perez-Riverol et al., 2022) partner repository with the dataset identifier PXD047830 the data were made public.

### 2.12 Data analysis

The Prism 6.07 program (GraphPad) was used for the statistical analysis and visualization. The experiments were analyzed for significance using *t*-test, Mann–Whitney test, or ANOVA with recommended multiple comparison posttests (as indicated in the results). Survival curves were compared by log-rank (Mantel-Cox) test. Differences were considered statistically significant at *p* < 0.05. All data generated or analyzed during this study are included in this published article.

## 3 Results

### 3.1 OMVs enhance *F. tularensis* entry into BMMs and suppress its intracellular proliferation

**Figure 1:**
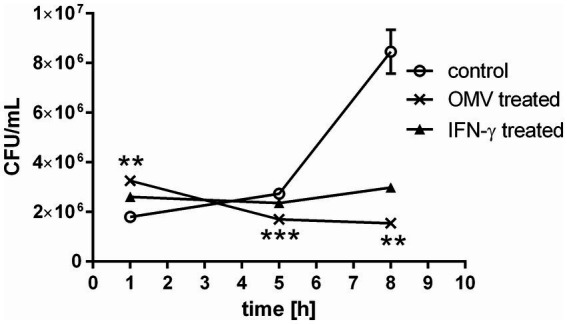
Bacterial proliferation in OMV-treated macrophages. BMMs were treated with OMVs or IFN-γ for 24 h and then infected with *F. tularensis* subsp. *holarctica* FSC200. The cells were lysed at 1, 5, and 8 h post-infection and the number of intracellular bacteria was enumerated by CFU plating. The data are expressed as mean ± SEM (*n* = 3), results shown are representative of five independent experiments. Statistical significance was determined by Holm-Sidak’s multiple comparisons test (**p < 0.01, ***p < 0.001).

Macrophages represent the primary host cell type for *Francisella*. The bacterium enters the BMMs within a few minutes, escapes the phagosome readily and proliferates exponentially in the cytosol within the first 24–48 h after infection (Clemens and Horwitz, 2007; Meibom and Charbit, 2010). The OMVs released from *Francisella* seem to be engaged in the entry mechanism of the bacteria into the macrophages (Pavkova et al., 2021). To get a closer knowledge of OMVs impact on macrophage encounters with *Francisella*, we examined the proliferation of *Francisella* in BMMs that were induced by OMVs 24 h prior to infection (Figure 1). At this time point, the cells were namely shown to respond to OMVs presence with a pro-inflammatory response as we demonstrated in our previous study (Pavkova et al., 2021). The entry of the bacteria into host cells was significantly enhanced in the OMV-treated BMMs compared to control as observed 1 h post-infection. After 5–8 h post-infection rapid intracellular multiplication was observed in the control macrophages. On contrary, OMVs pre-treatment suppressed the bacterial proliferation significantly, similarly as did the IFN-γ pre-treatment. It is known that IFN-γ induces pro-inflammatory activity in macrophages and suppresses the cytosolic replication of *Francisella* independently of known bactericidal mechanisms (Edwards et al., 2010; Wallet et al., 2017). In our experimental model, the effect of OMVs treatment resembled that of IFN-γ.

### 3.2 Infection by *Francisella* can partially suppress the pro-inflammatory effect of OMVs in macrophages

**Figure 2:**
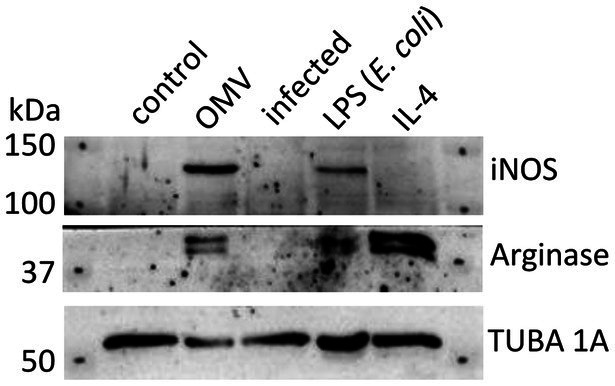
Western blot analysis of BMMs treated with OMVs, LPS from *E. coli*, IL-4, or infected by *F. tularensis* subsp. *holarctica* FSC200. Untreated macrophages were taken as control. Results are representative of two independent experiments.

Based on the results of earlier published studies (Bauler et al., 2014; Pavkova et al., 2021), the OMVs revealed pro-inflammatory potential on macrophages that was apparent from dose- and time-dependent elevation of inflammatory cytokines, including TNF-α, IL-6, IL-12p70, CXCL-1, MCP-1, and IL-1α. To closer characterize the phenotype of OMV-primed macrophages, we evaluated the production of iNOS and arginase 1 in comparison with BMMs stimulated either with *E. coli* LPS or IL-4. In general, LPS stimulates in macrophages the production of iNOS and promotes their M1 polarization, while IL-4 activates arginase synthesis as an indicative of the M2a phenotype (Arango Duque and Descoteaux, 2014). As can be seen from Figure 2 and Supplementary material S1, the OMVs induced the iNOS and to a lesser extent also arginase 1 productions, thus mimicking rather the effect of LPS and its M1 polarizing effect.

The known pro-inflammatory effect of OMVs on BMMs are in deep contrast with the ability of *Francisella* to actively inhibit the innate immune response (Bosio et al., 2007; Parsa et al., 2008; Dotson et al., 2013; Pavkova et al., 2021). The infection of BMMs with virulent strains of *F. tularensis* does not induce cytokine and chemokine release (Bauler et al., 2014; Pavkova et al., 2021). We were interested in whether the immunosuppressive effect of the bacterium can predominate over the passive but strong pro-inflammatory effect of the OMVs.

**Figure 3:**
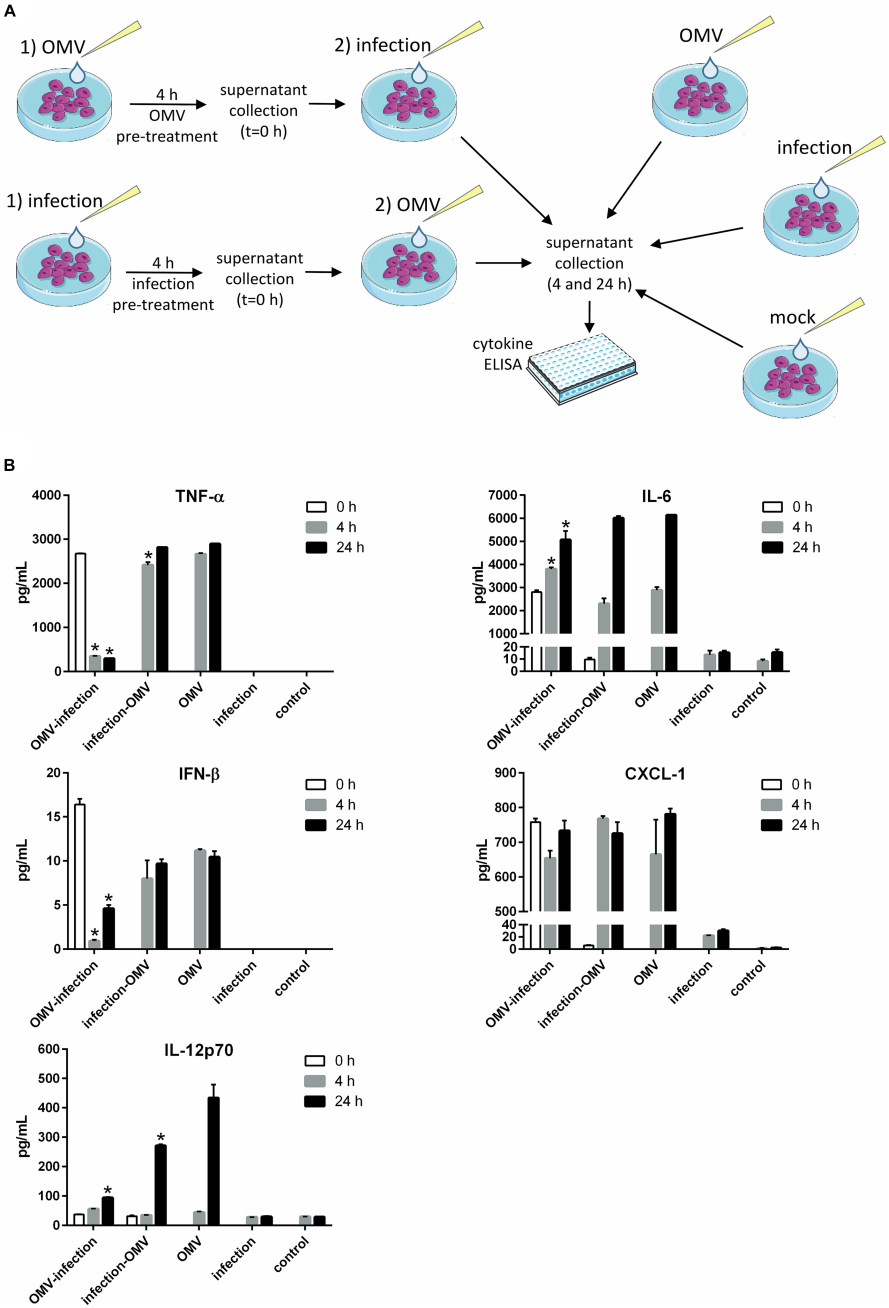
Cytokine production in macrophages treated sequentially with a combination of OMVs and infection by *Francisella*. **(A)** Experimental scheme: BMMs were pre-treated with OMVs or *F. tularensis* FSC200 for 4 h and the supernatant was collected for cytokine detection (*t* = 0 h). Sequentially BMMs were infected with FSC200 or treated with OMVs and incubated further 4 or 24 h for cytokine detection. Three independent controls were used—BMMs treated with OMVs only, BMMs infected only, and mock-treated BMMs. **(B)** Selected cytokines were determined in the culture supernatants by ELISA. The data are the mean ± SEM (*n* = 3), results shown are representatives of two independent experiments. Significance is shown for comparison versus the group treated only with OMVs in the respective time intervals (*p ≤ 0.05), two-way ANOVA followed by Dunnett’s multiple comparison *post hoc* test. The figure was partly generated using Servier Medical Art provided by Servier, licensed under a Creative Commons Attribution 3.0 Unported License.

For this purpose, we tested the secretion of selected cytokines in BMMs pre-treated with OMVs and then infected with the parent bacterium FSC200 and in BMMs treated in the opposite order: pre-infected with the bacterium and then treated with OMVs (the treatment scheme is in Figure 3A). The cytokines with the most prominent increase after OMVs treatment as observed previously (Pavkova et al., 2021) were selected for this analysis.

The infection decreased significantly the levels of TNF-α and IFN-β induced by OMVs pre-treatment. On the other hand, the OMVs were able to restore these cytokines secretion in infected cells (for 4 and 24 h) to the same extent as in non-infected cells. A similar effect was also observed for IL-12p70, but only 24 h post-treatment. At this time point, the OMVs induced secretion of this cytokine in contrast to the FSC200. In FSC200 pre-treated cells, the secretion of IL-12p70 was significantly elevated as well, although it did not reach the same levels as for OMVs alone. On the other hand, infection of OMVs pre-treated cells was able to suppress this effect. On the opposite, the strong stimulatory effect of OMVs on IL-6 and the chemokine CXCL-1 secretions always predominated the suppression induced by infection no matter whether BMMs were infected before or after OMVs treatment (Figure 3B).

### 3.3 The evaluation of OMVs effect in mice

#### 3.3.1 *In vivo* protective effect of OMVs against infection

**Figure 4:**
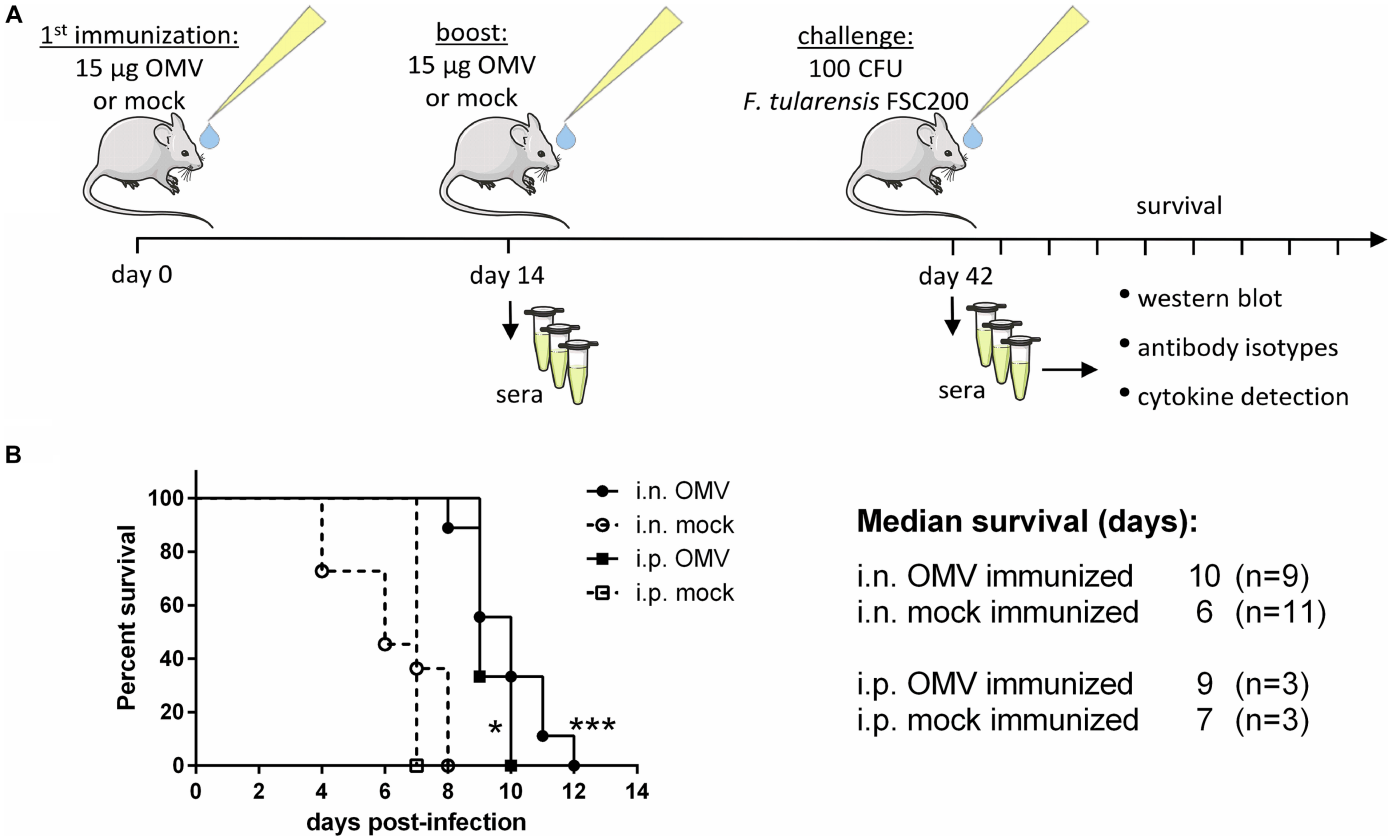
Protective effect of OMVs against infection of *F. tularensis* subsp. *holarctica* FSC200. **(A)** Vaccination and sera collection scheme: BALB/c mice were immunized i.n. or i.p. with 15 μg of isolated OMVs or mock and after 14 days they were boosted with the same. Six weeks after the first immunization the mice were challenged with 100 CFU of FSC200 per mouse and observed for survival. Sera were taken for the detection of cytokines, antibody isotypes, and immunoreactive proteins 14 and 42 days after immunization. **(B)** Survival curves after i.n. and i.p. administration, (*p < 0.05, ***p < 0.0001) significantly longer survival according to Mantel-Cox test. The figure was partly generated using Servier Medical Art provided by Servier, licensed under a Creative Commons Attribution 3.0 Unported License.

Mice were immunized i.n. or i.p. twice with the interval of 14 days and then challenged with the infection of *F. tularensis* subsp. *holarctica* FSC200 (in the same route of administration as the vaccination) 6 weeks after the first immunization to observe the survival, see the immunization scheme in Figure 4A. The median survival was significantly prolonged from 6 days to 10 in the OMV-vaccinated mice after i.n. administration and from 7 to 9 days in the i.p. administration (Figure 4B). After the second dose of OMVs the mice demonstrated strong symptoms similar to *Francisella* infection with pilo-erection and lethargy but they recovered within a few days.

#### 3.3.2 *In vivo* cytokine and humoral immune response in mice after OMVs vaccination

**Figure 5:**
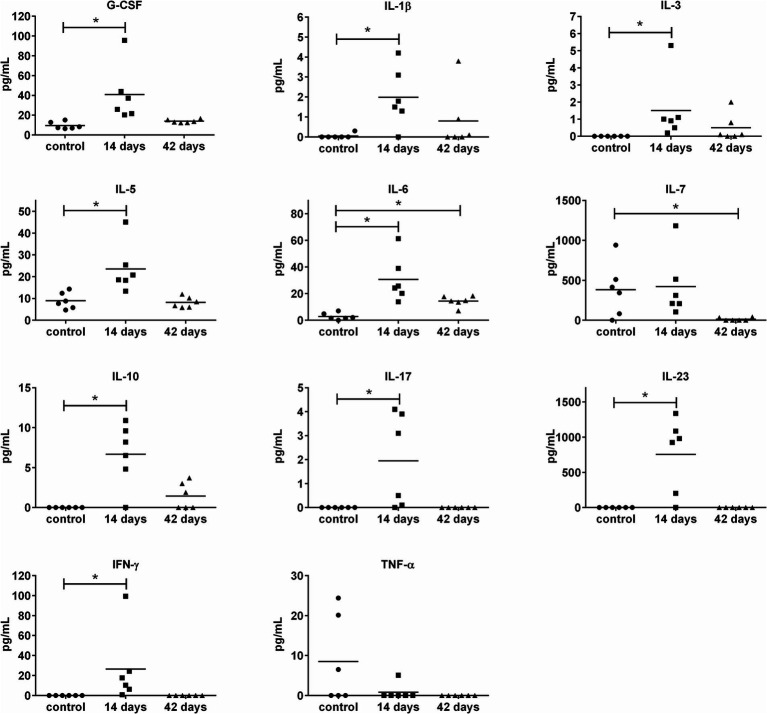
Cytokine levels in murine sera 14 and 42 days after i.n. immunization with OMVs. The immunization and sera collection scheme is depicted in Figure 4A. Cytokine levels were determined by a fluorescence-based multiplex Quantibody ELISA microarray chip. The data are shown as individual concentrations and mean (*n* = 6). Significance was estimated from the Mann–Whitney test (*p ≤ 0.05).

To get deeper insights into the immune response to the OMVs application, the set of 20 cytokines and 8 antibody isotypes were screened in sera collected from mice on days 14 and 42 post-i.n. vaccination. The levels of 10 cytokines changed in response to OMVs predominantly on day 14 post-treatment, followed by a decreasing trend on day 42 (Figure 5). There was an obvious trend in increased secretion of IL-1β, IL-6, IL-10, IL-5, IL-17, IL-23, G-CSF, IL-3, and IFN-γ on day 14 after the OMVs immunization compared to the mock-treated mice. Forty-two days after the OMVs treatment and 28 days after the boost, the amounts of almost all of these cytokines dropped down often up to the levels comparable with the control group. Surprisingly, the levels of TNF-α were not elevated in response to OMVs treatment.

**Figure 6:**
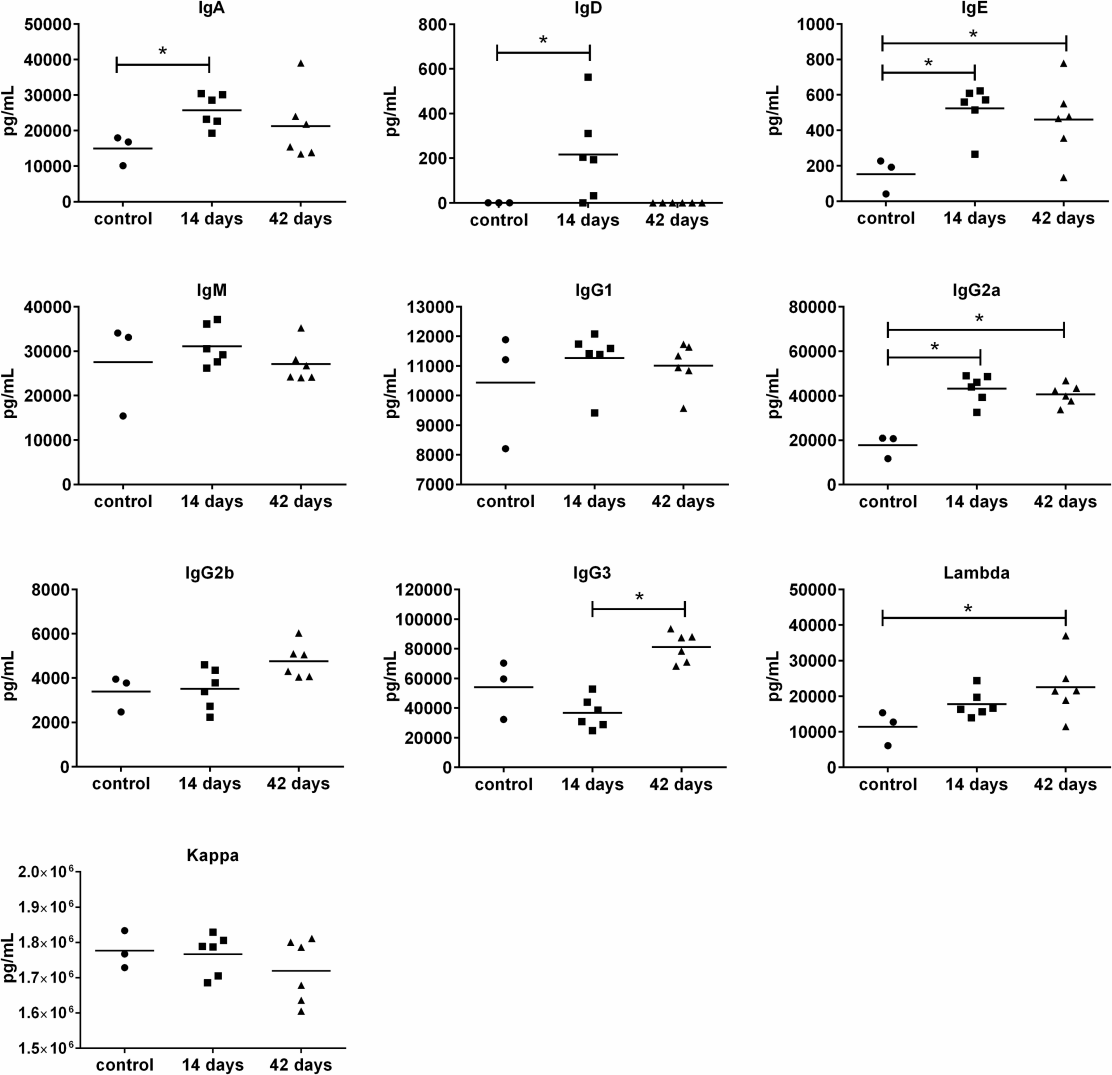
Antibody isotype levels in murine sera 14 and 42 days after i.n. immunization with OMVs. The immunization and sera collection scheme is depicted in Figure 4A. Antibody isotype levels were determined by fluorescence-based multiplex Quantibody Mouse Immunoglobulin Isotype Array. The data are shown as individual concentrations and mean (*n* = 6 in OMVs vaccinated and 3 in control). Significance was estimated from an unpaired *t*-test with Welch’s correction (*p ≤ 0.05).

For the humoral adaptive immune response (Figure 6), increased levels of IgE, IgG2a, and IgA antibodies were determined on day 14 and persisted even at day 42. For IgG3, only a late increase was observed (on day 42). Interestingly, most of the OMVs-treated mice (4 of 6) revealed increased levels of IgD on day 14. Taken together, OMVs immunization elicits unequivocal cytokine and antibody response in mice but does not provide satisfactory protection against subsequent infection with the virulent FSC200 strain.

#### 3.3.3 The sera from OMVs-immunized mice recognize known immunoreactive proteins

To further analyze the specific antibody response of mice immunized with OMVs, we used a classical immunoproteomic approach. In this strategy, 2D Western blots in a pH range of 3–10 were prepared from FSC200 whole cell lysates, that were then probed with sera pooled from 3 mice treated with OMVs i.n. or i.p. for 14 or 42 days according to the scheme shown in Figure 4A. The 2D Western blots with the detected immunoreactive proteins are shown in Supplementary material S2. The immunoreactive spots were manually aligned to protein spots on the corresponding silver-stained membrane and Coomassie blue-stained 2D gel examining also the archived reference 2D gels (Proteome 2D-PAGE Database, 2023) and subsequently analyzed using LC–MS/MS. In parallel, we also checked the reactivity of control sera from non-vaccinated mice. As seen in Supplementary material S2, several non-specific reactions were observed and these were excluded from further analysis. Only unambiguously assigned and identified protein spots are labeled with relevant FTS gene loci in the figures and they are also summarized in Table 1.

**Table 1:** Immunoreactive proteins detected in sera of mice vaccinated with OMVs.

Protein name gene locus^a^ *gene name*	i.n.		i.p.		Immunoreactive (previously observed—ref.)
	Day 14	Day 42	Day 14	Day 42	
Elongation factor Tu FTS_1709 (FTT_0137) *tuf*	+++^b^	+++		(+)	Janovská et al. (2007), Twine et al. (2010), Sharma et al. (2011)
Hypothetical protein FTS_0571 FTS_0571 (FTT_1539c)	+	++	+	++	Havlasová et al. (2005), Twine et al. (2006), Eyles et al. (2007), Janovská et al. (2007), Chu et al. (2014), Gaur et al. (2017)
OmpA family protein FTS_1295 (FTT_0583) *fopA1*	++	+++	++	+++	Havlasová et al. (2005), Eyles et al. (2007), Huntley et al. (2007), Janovská et al. (2007), Twine et al. (2010)
Malate dehydrogenase FTS_0967 (FTT_0535c) *mdh*	+	++			Havlasová et al. (2005)
Hypothetical protein FTS_1201 FTS_1201 (FTT_0975)	++	+++		++	Havlasová et al. (2005), Eyles et al. (2007), Sundaresh et al. (2007), Chu et al. (2014)
Intracellular growth locus protein C FTS_0099, FTS_1127 (FTT_1357c, FTT_1712c) *iglC1*, *iglC2*	(+)	(+)		+++	Havlasová et al. (2005), Twine et al. (2006), Gaur et al. (2017)
Outer membrane protein of unknown function FTS_0008 (FTT_1747) OmpA family peptidoglycan-associated lipoprotein FTS_0334 (FTT_0842)	+	+	+	+	Twine et al. (2006), Eyles et al. (2007), Golovliov et al. (2013), Chu et al. (2014)
Lipoprotein releasing system, subunit A, outer membrane lipoproteins carrier FTS_1661 (FTT_1636) *lolA*	+	++		+	Chu et al. (2014)
AhpC/TSA family protein FTS_0990 (FTT_0557)			++	+	Twine et al. (2006), Janovská et al. (2007)

a Locus tags and gene names from FSC200 (and corresponding SchuS4) database.

b The intensity of immunoreaction from no reactivity: empty cell, very weak reactivity: (+) to very intense reactivity: +++ for sera collected at day 14 or 42 after OMVs immunization by i.n. or i.p. routes.

The protein profiles detected on 2D immunoblots demonstrate, that the spectrum of antigens recognized by antisera differs depending on the time after immunization and also on the route of entry. On day 14 after i.n. administration of OMVs we were able to identify 9 unique proteins and most of them also persisted on day 42. The most intense reactivity was obvious for the elongation factor Tu (EF-Tu), OmpA family protein (FopA), and the hypothetical protein FTS_1201. Regarding the i.p. immunization, the antigen patterns were less pronounced and only 5 proteins could be identified on day 14, however, many more antigens were detected on day 42. As most of them were the same as for i.n. administration it can be deduced, that the humoral response after the i.p. administration is slightly delayed compared to the i.n. administration.

Interestingly, the i.p. route of administration evoked strong production of antibodies directed against the IglC protein on day 42, while only very weak reactivity was detected after i.n. administration in both intervals. Similarly, a spot corresponding to the AhpC/TSA family protein was detected only after i.p. administration. On the contrary, EF-Tu predominated, when the OMVs were applied i.n. All the identified proteins have previously been reported in the literature to be immunoreactive with *Francisella* antisera, they were found in the OMVs, and some of them were OMV-enriched (Klimentova et al., 2019). Several of them are well-known virulence factors including the IglC protein (Golovliov et al., 2003), AhpC/TSA family protein (Alharbi et al., 2019), or OmpA family lipoprotein (Hickey et al., 2011).

## 4 Discussion

OMVs derived from pathogenic bacteria are considered a promising tool for modulation of the host immune system to induce or at least reinforce the protection against infectious diseases as they can provide a variety of ideal antigenic determinants. However, it is also important to consider the presence of high concentrations of LPS and other TLR-activating molecules, that ensure the OMVs immunogenicity on one side, but on the other hand can induce detrimental inflammation response, limiting thus their clinical usage.

Previously, we demonstrated that OMVs derived from *F. tularensis* FSC200 enter macrophages by several mechanisms and the entry is partially influenced by the LPS O-antigen (Pavkova et al., 2021). Thereafter they exhibit an M1 polarizing effect on the macrophages forcing them to the production of predominantly pro-inflammatory cytokines. Despite this effect, OMVs had no demonstrable impact on cell cytotoxicity and viability (Pavkova et al., 2021). To follow up this study we further evaluated the impact of OMVs pre-treatment on macrophages infected with *F. tularensis*. The intracellular life cycle of *F. tularensis* consists of rapid escape from phagosome followed by extensive replication in host cell cytosol resulting in cell death and reinfection of neighboring cells by about 20 h post-infection. The viability of the bacterium and its ability to escape to the cytosol is then essential for the inhibition of the innate immune pathways and complete evasion of immune system surveillance (Putzova et al., 2017). *Francisella* actively interferes with the host cell response on multiple levels, e.g., by alteration of its surface, its gene expression, post-translational modifications, secretion of proteins, modification of host structures, or regulation of host gene expression (Hazlett et al., 2008; Pavkova et al., 2013; Fabrik et al., 2018). On the other hand, isolated OMVs are considered to be a “non-viable mixture of *Francisella* antigens” separated from the bacterial surface, their interaction with the host cell is rather passive and might be compared to the effect of the inactivated bacterium. The pre-activation of macrophages by OMVs promoted *F. tularensis* entry, but the cytosolic replication was nearly inhibited during the first 8 h post-infection. The ability of cells to control the bacterial replication seems to be partially mediated by IL-6 and CXCL-1, the murine functional homolog of IL-8, as the bacterium was not able to fully reverse their production induced by the OMVs. The IL-6 has previously been shown to be important for host resistance to *F. tularensis* LVS infection (Kurtz et al., 2013).

The OMVs effect on bacterial intracellular replication was comparable to that of IFN-γ. IFN-γ is a potent inducer of polarization of macrophages to M1 phenotype with predominantly bactericidal function and it is widely known to restrict *F. tularensis* cytosolic growth most likely in the guanylate-binding protein (GBP)-dependent manner (Edwards et al., 2010; Wallet et al., 2017). The GBPs were found to act as regulators of OMV-mediated inflammation in macrophages treated with OMVs derived from *E. coli*. Through the LPS, the GBPs can target the intracellularly localized OMVs, which leads to inflammasome activation (Santos et al., 2018). It remains questionable, whether a similar mechanism might also participate in *F. tularensis* OMVs’ pro-inflammatory character, as *F. tularensis* LPS has an atypical structure making it less toxic and immunogenic and unable to stimulate neither TLR4 nor TLR2 signalization (Barker et al., 2006; Dueñas et al., 2006). Other TLR-activating ligands present in OMVs (e.g., Tul4, DsbA, or the hypothetical protein FTT_1416c) were previously found to be highly enriched in the OMVs (Klimentova et al., 2019) but their exact role in the interaction or protection remains to be investigated.

Other studies on bacteria with intracellular life cycle mostly came to the conclusion that OMVs are able to render macrophages less responsive to infection promoting thus pathogenesis and spread of bacteria through the host organism. OMVs from *Brucella abortus* were found to enhance the adhesion and internalization of bacteria by human monocytes and decrease the pro-inflammatory cytokine response in cells infected subsequently with the parent bacterium (Pollak et al., 2012). Membrane vesicles from *Mycobacterium bovis* were shown to induce bacterial dissemination in pre-treated mice (Prados-Rosales et al., 2011). Human THP-1 macrophages pre-treated with OMVs derived from *Legionella pneumophila* showed reduced bacterial replication, but only within the first 24 h post-infection. In later time intervals, the OMVs revealed an enhancing effect on bacterial load together with decreased induction of pro-inflammatory cytokines (Jung et al., 2016). In our study, we deliberately omitted the analysis of bacterial proliferation in later post-infection time, when cell death followed by secondary infection can occur which might distort the primary effect of OMVs.

Two previously published studies demonstrated the immunoprotective effect of OMVs derived from *F. novicida* against subsequent infection with *F. novicida* in mice (Pierson et al., 2011; McCaig et al., 2013), however, the results obtained from experiments using *F. novicida* should be interpreted with caution. *F. novicida* is closely related to *F. tularensis*, but the two species significantly differ in their virulence. While it is non-pathogenic in healthy humans, it remains highly virulent in mice, causing tularemia-like disease. As the manipulation with this species does not require such strict safety precautions, it is often used as a laboratory surrogate for *F. tularensis*. Here, we investigated the effect of OMVs derived from a pathogenic strain of *F. tularensis* on subsequent challenge with the same strain in a mouse model. Concurrently, we explored the cytokine and humoral immune response induced by the OMVs vaccination. In our experimental model, the OMVs were not able to induce a satisfactory immunoprotective effect against subsequent infection, as we observed only a few days of prolonged survival.

The OMVs alone evidently induced a systemic inflammatory reaction particularly after the administration of the booster dose, when the mice developed typical symptoms of inflammation. The inflammatory response was further corroborated by the increased levels of pro-inflammatory cytokines, like IL-1β, IL-6, IL-23, and IFN-γ. The cytokines IFN-γ and IL-6 have previously been identified as critical for the clearance of *F. tularensis* (Elkins et al., 2007; Edwards et al., 2010; Cowley and Elkins, 2011; Kurtz et al., 2013; Steiner et al., 2014). Nevertheless, TNF-α, another inflammatory marker essential for bacterial elimination and critical for the survival of infected mice (Elkins et al., 2007), was not upregulated in response to OMVs in the *in vivo* model, even though significant elevation was observed in isolated macrophages as shown in this study and previously (Pavkova et al., 2021). This observation might be the consequence of elevated production of IL-10, which acts as a potent inhibitor of TNF-α production (Armstrong et al., 1996). Due to its anti-inflammatory activities, IL-10 plays an important role in regulating the acute inflammation response within safe limits for the host organism. On the other hand, its production can lead to ineffective pathogen clearance and persistent infection which was also demonstrated in *F. tularensis* (Armstrong et al., 1996; Duell et al., 2012; Metzger et al., 2013). The pro-inflammatory potential of OMVs is well-known and not entirely surprising because they contain a number of inflammatory substances as demonstrated in animal models with various bacteria (Chen et al., 2023). The inflammation-inducing OMVs components are necessary for the immunoprotectivity, but their effect may be highly detrimental to the organism, as well. The problem with setting a very fine balance between the pro-inflammatory potential and immunoreactivity limits the usage of OMVs as an effective and safe vaccine.

As *F. tularensis* is an intracellular pathogen, not only humoral but also cell-mediated immunity is needed for the induction of successful protection against infection. OMVs vaccination induced a broad spectrum of cytokines related to the development or activity of Th1 (IL-7, IFN-γ), Th2 (IL-5, IL-6), and also Th17 (IL-17, IL-23) in most of the vaccinated animals. From those cytokines especially IFN-γ was identified in a number of animal models to be crucial for protection against tularemia as summarized in Elkins et al. (2007). IL-23 is known to positively regulate IFN-γ production. In addition, it also augments IL-10 release and induces IL-17 synthesis. Nevertheless, Kurtz et al. found IL-23 dispensable for immunity to LVS strain (Kurtz et al., 2014). IL-17 is able to stimulate the production of IL-6. Both these cytokines, IL-17 and IL-6, were demonstrated to play a role in the progression of primary infection with LVS, but not against LVS secondary challenge or primary challenge with virulent *F. tularensis* (Kurtz et al., 2013; Laws et al., 2013; Skyberg et al., 2013). The IL-5 is important for the development of B1 cells and it promotes the production of IgM in response to immunization with *F. tularensis* LPS (Barbosa et al., 2021). However the levels of IgM were not increased after OMVs immunization compared to the non-vaccinated control group.

The most robust humoral adaptive immune response induced by OMVs concerned IgG2a production. After the boost vaccination, levels of IgG3 were also increased. In mice, the switch to IgG2a and IgG3 is stimulated by IFN-γ and it is indicative of the Th1 profile. The IgG2 and IgG3 subclasses are also produced during tularemia infection in humans (with the predominance of IgG2) (Rastawicki et al., 2014). Elevated production of IgG2a and IgG3 was repeatedly observed in response to vaccination with membrane fractions isolated from *F. tularensis* (Huntley et al., 2008; Sutherland et al., 2012). Induction of IgG3 was also reported in mice immunized with *F. tularensis* LPS (Cole et al., 2011). In contrast to OMVs, the membrane fractions additionally induced higher titers of IgG1 and IgM antibodies associated with Th2 response. Huntley et al. (2008) even detected elevated amounts of IgA, that play an important role in mucosal immunity and are suggested to be involved in protection to pulmonary *F. tularensis* LVS infection (Furuya et al., 2013). In our study, IgA antibodies were only slightly increased after the first vaccination but not significantly after the boost. So even though the OMVs contain a large spectrum of proteins inclusive of membrane proteins, they seem not to be able to induce such a broad humoral response as the isolated membrane fractions. This might be reflected in the more pronounced protective effect of *F. tularensis* membrane fractions, demonstrated in the above-mentioned studies (Huntley et al., 2008; Sutherland et al., 2012; Post et al., 2017). Nevertheless, it should be noted, that the membrane fractions were administered in combination with adjuvants (Huntley et al., 2008) or even encapsulated into a nanoparticulate delivery system together with an adjuvant (Post et al., 2017) to strengthen the immune response. It is also important to mention that OMVs are highly enriched in some proteins when compared to the membrane fraction, in some of the proteins the enrichment is in the order of 100× (Klimentova et al., 2019). Several of them were strongly recognized by the sera of the immunized animals and their role in the protective effect as well as in the systemic inflammatory response is yet to be explained.

Taken together, the BALB/c mice were able to mount quite a broad immune response to OMVs administration, that was, however, not sufficient to fully protect them against the FSC200 challenge. There are several indicators for this failure. So far, the most important cytokines identified as essential for effective protection are IFN-γ and TNF-α. The levels of IFN-γ were, however, upregulated only moderately, and after the first vaccination, later it was at the control level. Concerning TNF-α, no upregulation was detected in our infection model at all.

Sera from mice immunized with OMVs recognized a set of previously described immunoreactive *F. tularensis* proteins. Immunogens prevailing after i.n. OMV vaccination included EF-Tu, OmpA family protein, and hypothetical proteins FTS_1201 and FTS_0571, while the most dominant protein detected by sera from i.p. vaccinated mice was IglC. The OmpA family protein (also known as FopA1 or FopA) together with EF-Tu were identified as primary candidates for a defined post-exposure vaccine (Chandler et al., 2015). The OmpA family proteins have been extensively studied by others in relation to immunogenicity. Mice immunized with recombinant FopA or FopA-specific antibodies were protected against lethal challenge with *F. tularensis* LVS, but not against the virulent SchuS4 strain (Hickey et al., 2011). EF-Tu, another known highly immunoreactive *F. tularensis* protein, was shown to elicit the production of inflammatory cytokines (IL-6, TNF-α) in a TLR4-dependent way (Sharma et al., 2011). Interestingly, several differences in the profiles due to the route of vaccination were detected in our study. From the above-mentioned antigens, EF-Tu and hypothetical protein FTS_1201 revealed much lower immunoreactivity with sera after i.p. administration. On the other hand, one of the most immunodominant spots corresponding to the IglC protein was very weak in the case of i.n. vaccination. IglC is encoded by a gene of *Francisella* pathogenicity island and it forms the tube of the type VI secretion system (Clemens et al., 2018). The peroxiredoxin from the AhpC/TSA family is another antigen recognized only by sera from i.p. vaccinated mice. As a key antioxidant enzyme, this protein contributes to oxidative and nitrosative stress resistance in virulent *F. tularensis* SchuS4 (Alharbi et al., 2019). The disparities in immunoproteomes indicate, that the different administration routes of the same antigen activate distinct humoral immune responses, which might have an impact on final vaccine effectivity (Nicol et al., 2021).

In summary, OMVs isolated from *F. tularensis* FSC200 induce quite a strong immune response on a cellular level as well as *in vivo* in mice. On the other hand, their protective effect, while significant, was not quite satisfactory regardless of the route of administration. Nevertheless, the response of the immune system, especially on the humoral level, indicates that the vesicles present an interesting source of immunoreactive material and their protective potential should be further evaluated. As in other bacteria, where vaccination with OMVs was more successful, also in *Francisella* the future protective studies should consider all the possibilities of the OMVs contents manipulation – either genetically or on the level of their isolation. Furthermore, the formulation of OMVs with suitable adjuvants to induce cell-mediated immunity should also be taken into consideration.

## Data availability statement

The data presented in the study are deposited in the ProteomeXchange Consortium via the PRIDE partner repository https://www.ebi.ac.uk/pride/; accession number PXD047830.

## Ethics statement

All experiments on mice were conducted under the supervision of the institution’s Animal Unit and were approved by the Animal Care and Use Committee of the Military Faculty of Medicine, University of Defence, Hradec Kralove, Czech Republic under project number 5/21. The study was conducted in accordance with the local legislation and institutional requirements.

## Author contributions

IP: Conceptualization, Investigation, Methodology, Visualization, Writing – original draft. JB: Formal analysis, Investigation, Methodology, Writing – original draft. KK: Investigation, Methodology, Writing – original draft. JS: Conceptualization, Funding acquisition, Supervision, Writing – review & editing. JK: Conceptualization, Formal analysis, Investigation, Methodology, Project administration, Supervision, Visualization, Writing – original draft, Writing – review & editing.

## Supplementary material

Supplementary Material 1: Western blot quantification of proteins from Figure 2.

Supplementary Material 2: Western blots with the detected immunoreactive proteins.

## Glossary

OMVs: Outer membrane vesicles
LPS: Lipopolysaccharide
TLR: Toll-like receptors
LVS: Live vaccine strain
BHI: Brain heart infusion
BMMs: Bone marrow macrophages
DMEM: Dulbecco’s modified Eagle medium
MOI: Multiplicity of infection
PBS: Phosphate buffered saline
PVDF: Polyvinylidene difluoride
TUBA 1A: Alpha tubulin 1A
iNOS: Inducible NO synthase
HRP: Horse radish peroxidase
i.n.: Intranasal
i.p.: Intraperitoneal
ABC: Ammonium bicarbonate
TBS: Tris-buffered saline
ACN: Acetonitrile
LC–MS/MS: Liquid chromatography and tandem mass spectrometry
SEM: Standard error of the mean
EF-Tu: Elongation factor Tu
GBP: Guanylate-binding protein
