# Supplementary material for: Protective potential of outer membrane vesicles derived from a virulent strain of Francisella tularensis
Source: Front Microbiol. 2024 Mar 12;15:1355872. doi: 10.3389/fmicb.2024.1355872 (PMC10963506; doi:10.3389/fmicb.2024.1355872)
Supplement: Supplementary Material 1: — Western blot quantification of proteins from Figure 2. [file Image_1.PDF]

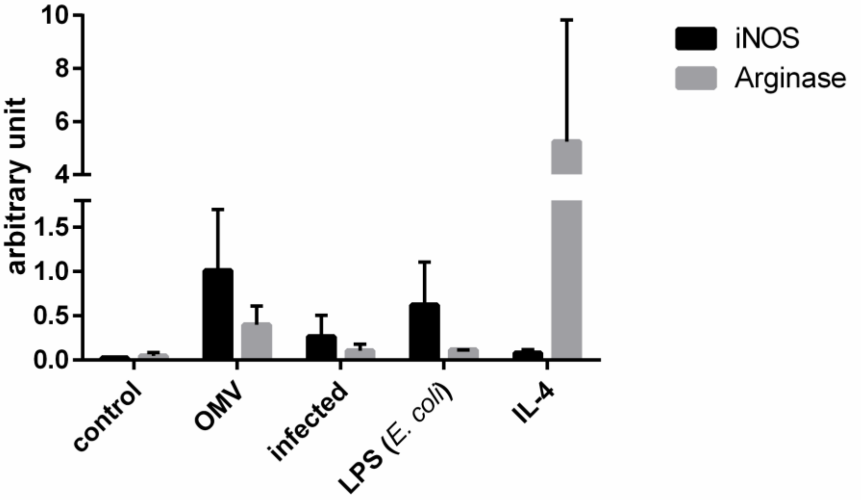

Supplementary Material 1. Western blot quantification of proteins from Figure 2. The quantification was performed with iBright Analysis Software (Thermo Fisher Scientific). Local background corrected volumes were used and the values were normalized using alpha tubulin 1A bands as the house-keeping protein. The data are presented as mean ± SEM of two distinct experiments.
