# Supplementary material for: Protective potential of outer membrane vesicles derived from a virulent strain of Francisella tularensis
Source: Front Microbiol. 2024 Mar 12;15:1355872. doi: 10.3389/fmicb.2024.1355872 (PMC10963506; doi:10.3389/fmicb.2024.1355872)

Supplementary Material 2: 2D Western blots with the detected immunoreactive proteins.

Whole cell lysate of FSC200 separated by 2D SDS-PAGE was used as the antigen for the detection of immunoreactive proteins recognized by the murine immune sera. The lysate (100 µg) was separated by isoelectric focusing on 7cm gradient pH 3-10 Immobiline DryStrip gels and 12% SDS-PAGE. The gels were electroblotted onto PVDF membrane. Immunoreactive proteins on the membranes were detected by control or immune sera (14 or 42 days post vaccination with OMV) pooled from three mice, polyclonal HRP-conjugated goat anti-mouse immunoglobulins were used for secondary antibody detection. Each immunoblot experiment was conducted in technical duplicate (left and right membrane on the images) and the membranes post detection were silver stained. Coomassie G-250 staining was used to visualize proteins on the gels for the purpose of mass spectrometry.
Protein spots on the membranes are labeled with FTS gene loci numbers (see the table below for the protein and gene names and for corresponding FTT numbers).

FTS locus	FTT locus	Protein name	Gene name
FTS_1709	FTT_0137	Elongation factor Tu	*tuf*
FTS_0571	FTT_1539c	Hypothetical protein FTS_0571	
FTS_1295	FTT_0583	OmpA family protein	*fopA1*
FTS_0967	FTT_0535c	Malate dehydrogenase	*mdh*
FTS_1201	FTT_0975	Hypothetical protein FTS_1201	
FTS_0099, FTS_1127	FTT_1357c, FTT_1712c	Intracellular growth locus protein C	*iglC1*, *iglC2*
FTS_0008	FTT_1747	Outer membrane protein of unknown function	
FTS_0334	FTT_0842	OmpA family peptidoglycan-associated lipoprotein	
FTS_1661	FTT_1636	Lipoprotein releasing system, subunit A, outer membrane lipoproteins carrier	*lolA*
FTS_0990	FTT_0557	AhpC/TSA family protein	

Intranasal immunization **repl. 1** **repl. 2**

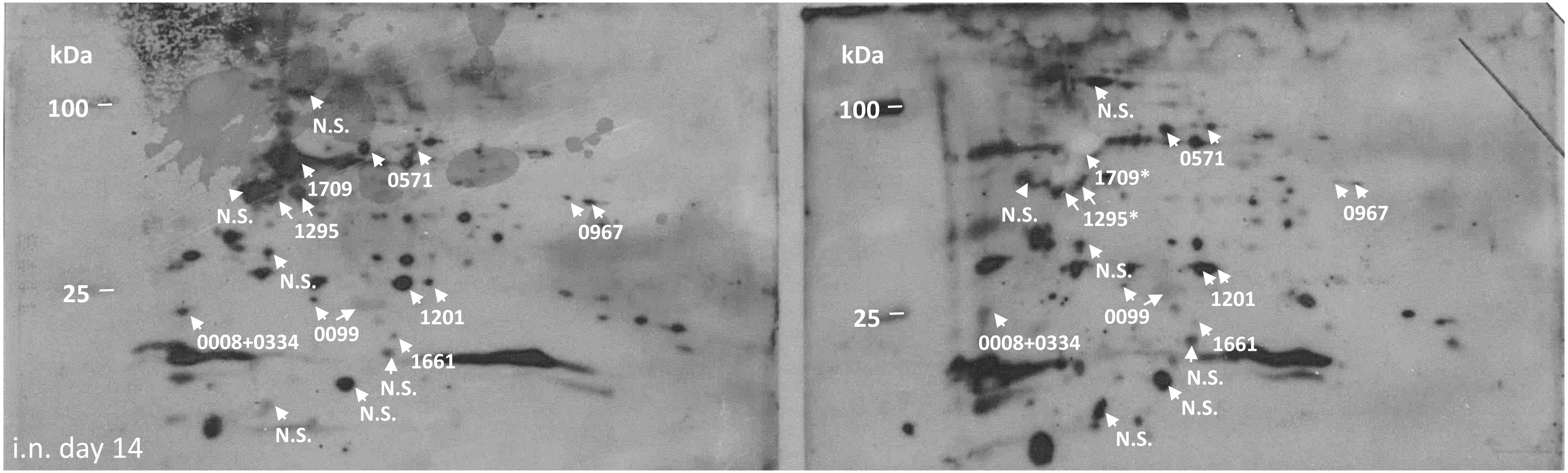

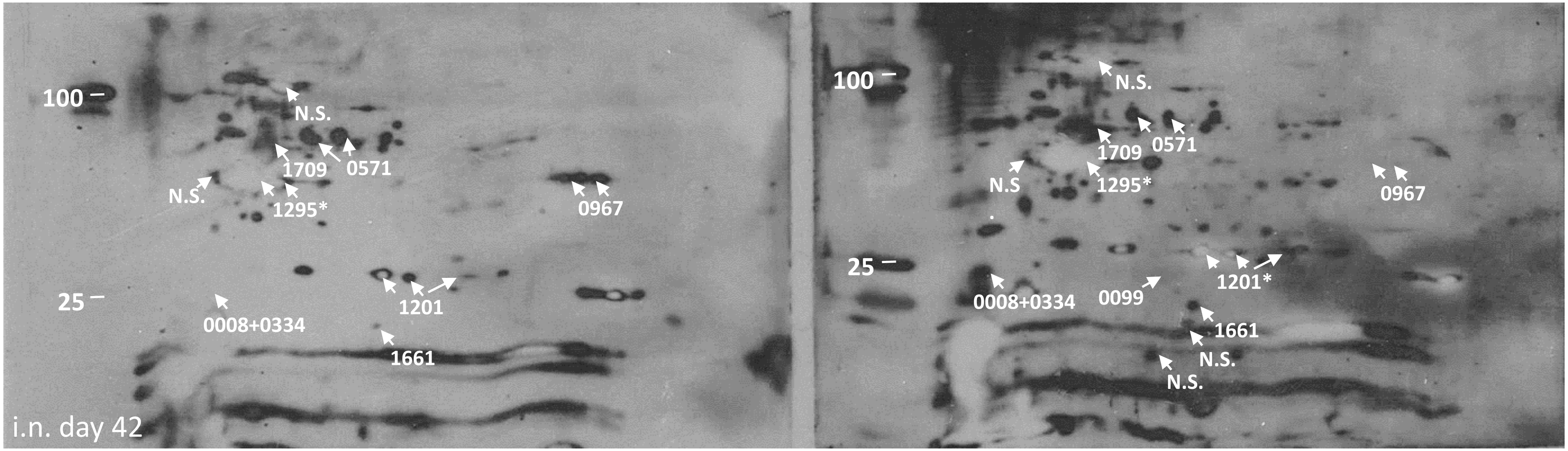

N.S. – non specific detection (as assigned from the membrane detected with naïve sera).

* – the spot shows white due to too strong reaction, the position was checked on shorter exposure images

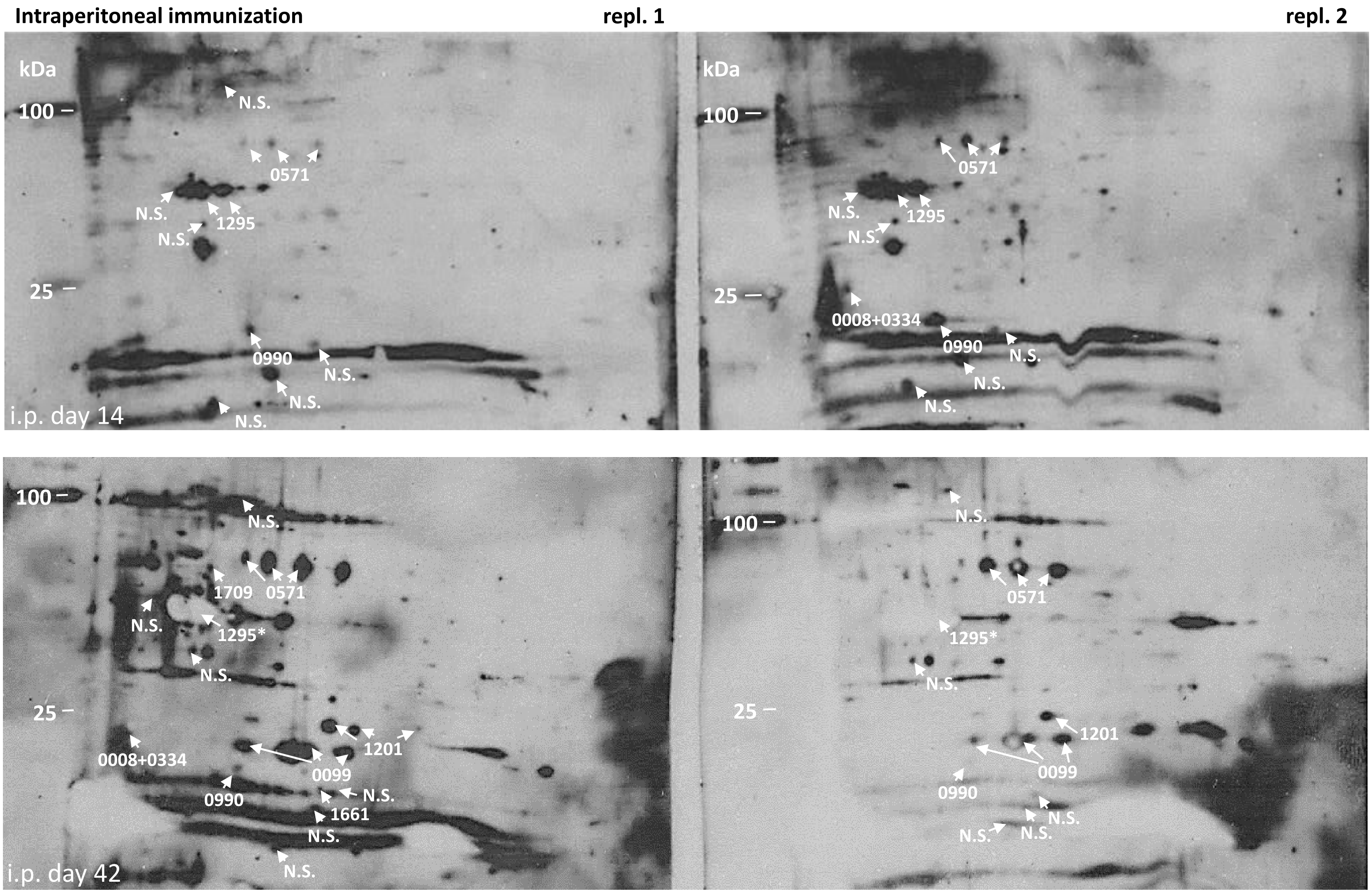

N.S. – non specific detection (as assigned from the membrane detected with naïve sera).

* – the spot shows white due to too strong reaction, the position was checked on shorter exposure images

Naïve sera (non specific detection)

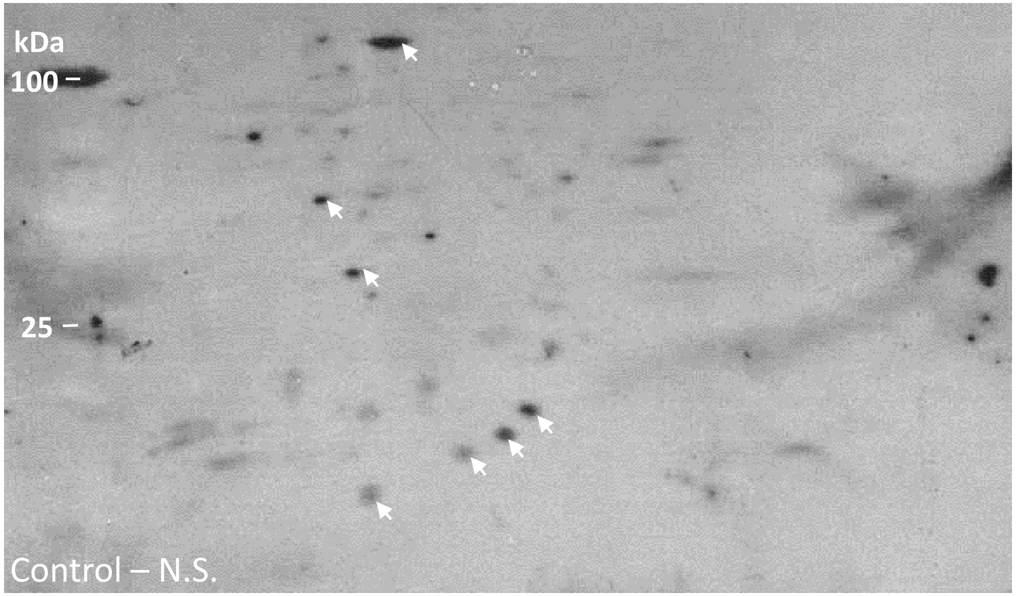

Coomassie G-250 stained gel, spots identified by mass spectrometry

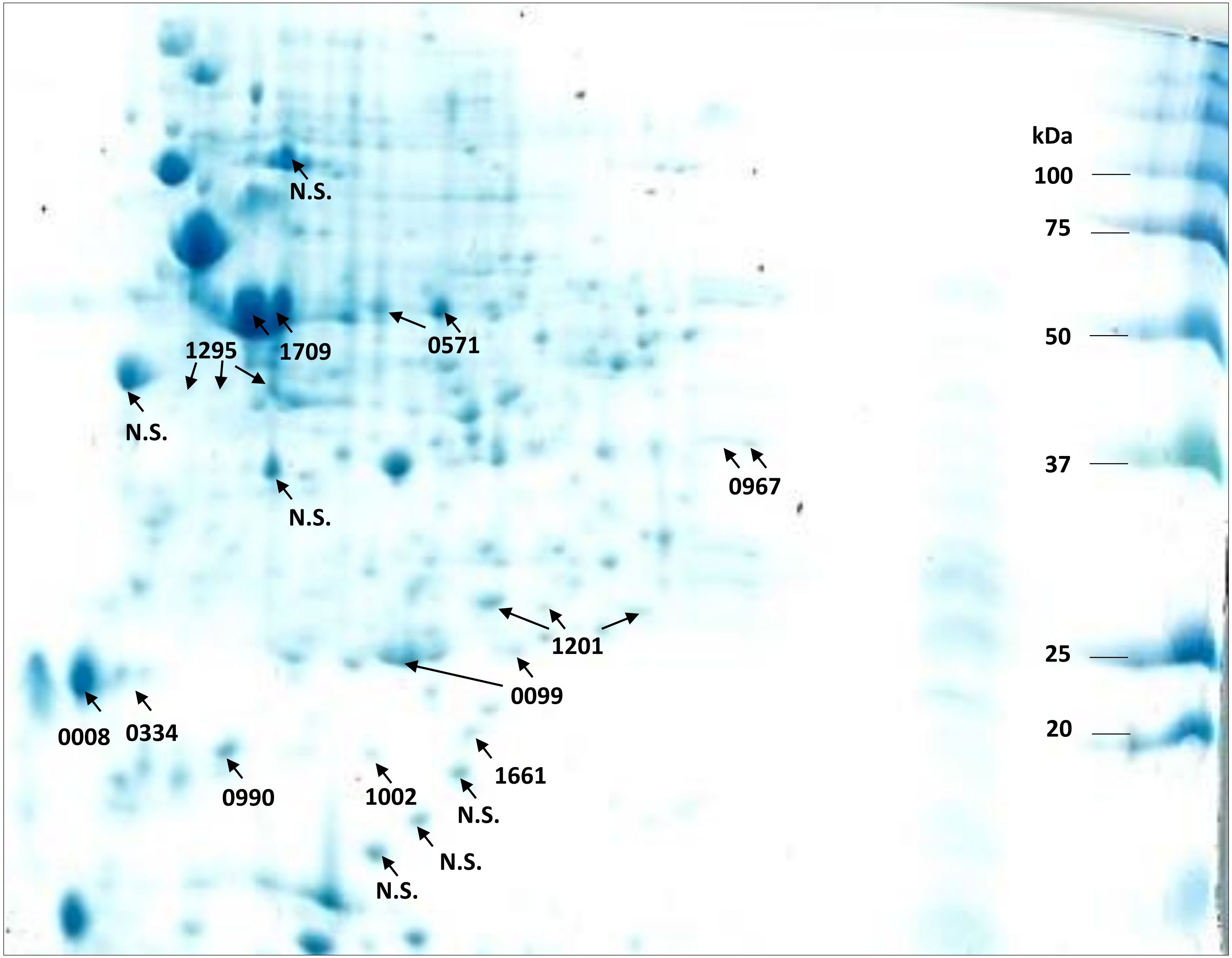

Supplement: Supplementary Material 2: — Western blots with the detected immunoreactive proteins. [file Image_2.PDF]
